# The Oxidation and Corrosion Resistance of AlCrNbSiTiN Multi-Principal Element Nitride Coatings

**DOI:** 10.3390/ma18204663

**Published:** 2025-10-10

**Authors:** Zhenbo Lan, Jiangang Deng, Heng Xu, Zhuolin Xu, Zhengqi Wen, Wei Long, Lei Zhang, Ruoxi Wang, Jie Liu, Yanming Chen

**Affiliations:** 1 State Grid Electric Power Research Institute, Wuhan NARi Co., Ltd., Wuhan 430072, China; 2 School of Power & Mechanical Engineering, Wuhan University, Wuhan 430072, China

**Keywords:** multi-principal element nitride coatings, arc ion plating, mechanical properties, high-temperature resistance, thermal stability, corrosion resistance

## Abstract

Multi-principal element nitrides have great application potential in protective coatings. However, the investigation of the oxidation and corrosion resistance of multi-principal element nitride coatings is still insufficient. The synthesis and high-temperature performance of AlCrNbSiTiN multi-principal element nitride coatings fabricated through optimized arc ion plating (AIP) were explored. Leveraging the high ionization efficiency and ion kinetic energy characteristic of AIP, coatings with significantly fewer internal defects were obtained. These coatings demonstrate superior mechanical properties, including a maximum hardness of 36.5 GPa and critical crack propagation resistance (CPR) values approaching 2000 N^2^. Optimal coatings exhibited exceptional water vapor corrosion resistance (5.15 at% O after 200 h). The coatings prepared at −150 V had the optimal corrosion resistance, with the coating resistance and corrosion current density being 1.68 × 10^4^ Ω·cm^2^ and 0.79 μA·cm^−2^, respectively. AlCrNbSiTiN coatings produced under these optimized AIP conditions exhibit remarkably high-temperature oxidation, highlighting their potential for use in demanding engineering applications.

## 1. Introduction

With the increasing demand for high-performance protective coatings in extreme environments, such as cutting tools, transition metal nitride coatings have been widely used due to their superior hardness, wear resistance, and thermal stability [1,2,3]. Transition metal nitride coatings, particularly those based on TiN and CrN architectures, have been extensively employed as protective layers for cutting tools and aerospace components due to their exceptional hardness and oxidation resistance [4,5,6]. However, conventional binary/ternary nitrides exhibit inherent limitations in thermal stability above 800 °C, where phase decomposition and grain coarsening lead to rapid property degradation [7,8].

To address this, high-entropy nitride coatings (HENs) have emerged as a revolutionary material [9,10]. Leveraging four core effects, HENs stabilize single-phase solid solutions, enabling superior high-temperature performance [11,12,13]. Huang et al. [14] investigated the thermal stability of (AlCrTiNbTa)N coatings under extreme conditions. The slow diffusion effect inhibited the high-temperature mutual diffusion of coating elements and delayed softening. The hardness of the coatings is 35.2 GPa at room temperature, and it can still maintain 24.1 GPa at 1000 °C. The (TiHfZrNbV)N coatings were prepared by reactive magnetron sputtering, with a hardness of 33.6 GPa [15]. The lattice distortion caused by the high-entropy effect significantly hinders dislocation slip and enhances the ability to resist plastic deformation.

However, the deposition of defect-free HENs remains challenging. Conventional physical vapor deposition (PVD) techniques often suffer from intrinsic defects such as droplets and porosity, limiting coating density and high-temperature resilience [16,17,18]. Arc ion plating (AIP) technology offers advantages in depositing dense nitride coatings through high ionization rates and kinetic energy bombardment, yet optimizing bias voltage remains critical to suppress macroparticle defects and residual stress [19,20]. Uncontrolled bias voltages during AIP induce deleterious effects. Low bias promotes macroparticle incorporation and porous growth, while excessive bias generates residual tensile stress through atomic resputtering.

Among HEN systems, Al-Cr-Nb-Si-Ti-N quinary coatings demonstrate outstanding potential owing to synergistic strengthening from refractory elements (Nb, Ti) and oxidation-resistant constituents (Al, Cr) [21,22]. However, there is a lack of research on its high-temperature resistance, oxidation behavior, and corrosion resistance. In this study, AlCrNbSiTiN HEN coatings were synthesized by AIP under controlled bias voltages. The microstructure of the coatings is regulated by controlling the bias voltages to enhance their mechanical properties and high-temperature resistance. The main objective was to fabricate hard coatings that could exhibit favorable oxidation and corrosion resistance and could be used at elevated temperatures. The interaction among bias voltages, coating densification, mechanical properties, and high-temperature stability was systematically studied. The principle of the high-temperature stability of the coatings and their thermal hardness was revealed. The effect of bias voltages on the structure and corrosion resistance of (AlCrNbSiTi)N coatings was displayed, providing significant scientific support for industrial applications, such as the production and performance improvement of protective coatings.

## 2. Experimental Details

### 2.1. Coatings Preparation

The coatings were synthesized utilizing AIP with varied bias voltages. A circular HEA target (99.99% purity) AlCrNbSiTi with a diameter of 150 mm was prepared by the sintering method with atomic ratios of 22:34:11:11:22. The 150 mm targets of the Al_22_Cr_34_Nb_11_Si_11_Ti_22_ alloy and pure Cr (99.99%) were used in the deposition process. Substrates (304 stainless steel, Si, and cemented carbide) underwent sequential ultrasonic cleaning in acetone and ethanol (15 min each). 304 stainless steel was mechanically polished to a roughness of approximately 10 μm. The target and substrate were 150 mm apart. Before deposition, the vacuum chamber was heated to 150 °C and vacuumed to below 3 × 10^−3^ Pa. Ar plasma etching (50 V bias, 90 A target current, 0.5 Pa) purified surfaces for 30 min. Subsequently, nitrogen was introduced to maintain the pressure of the chamber at 3.5 Pa. A Cr/CrN interlayer was deposited (70 A, N_2_:Ar = 1:1, 1.5 Pa) to enhance adhesion. AlCrNbSiTiN coatings were then synthesized at 70 A for 45 min under bias voltages (0–200 V).

### 2.2. Coatings Characterization

The Scanning electron microscopy (TESCAN, TESCANBrno, Ltd., Oxford, UK) and Atomic force microscopy (Shimadzu, SPM-9700HT, Kyoto, Japan) were used to observe morphology, and its composition was determined by the energy dispersive spectrophotometer (X-MAXN, Oxford, UK). The phase of the coatings was obtained by X-Ray Diffractometer (PANalytical, XPert Pro, Xi’an, China) with Cu Kα radiation, scan speed of 2°/min, and 2θ range from 20 to 80°. The nano-indenter (KLA, G200, Milpitas, CA, USA) equipped with the Berkovich indenter was employed to obtain the hardness and elastic modulus [23]. The test was performed using the continuous stiffness method and averaged over six measurements. The friction and wear tests were conducted using a ball-on-disk tribometer (HuaHui, MS-T300, Lanzhou, China) with a 304 stainless steel counterpart ball, load of 5 N, friction radius of 3 mm, rotation speed of 200 rpm, and test time of 30 min. The Hertzian contact stress is 0.385 MPa. The scratch toughness of coatings was evaluated using a scratch tester (HuaHui, MFT-4000, Lanzhou, China) under a loading rate of 100 N/min, load of 100 N, and scratch length of 10 mm. The electrochemical performance test of the coatings was conducted using a potentiostat (ELDY/CS-350, Beijing, China) in a 3.5 wt.% NaCl solution. The test adopts a three-electrode system, with a Pt sheet as the counter electrode, Ag/AgCl as the reference electrode, and the coated sample as the working electrode. Electrochemical impedance spectroscopy (EIS) tests and polarization curve tests were, respectively, conducted on the coatings. In the EIS test, frequency values range from 10^−5^ to 10^−2^ Hz, and the amplitude was 0.01 V. In the polarization curve test, the scanning rate was set at 0.1 mV/s. The coating test area is controlled at 1 cm^2^. The oxidation experiment was conducted in a horse boiler furnace, with an oxidation temperature of 600–900 °C, a heating rate set at 3 °C/min, and a holding time of 2 h.

## 3. Results and Discussion

### 3.1. Morphology and Composition

**Figure 1:**
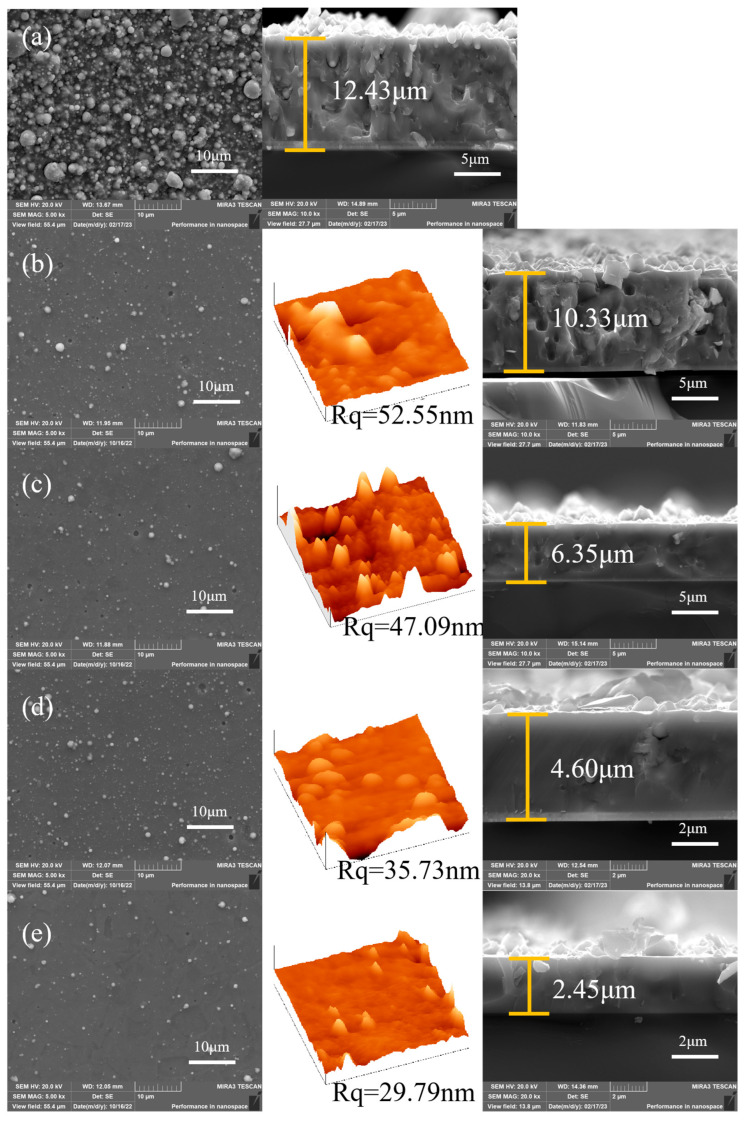
The surface, AFM, and cross-sectional morphology of the AlCrNbSiTiN coatings with different bias voltages. (**a**) 0 V, (**b**) −50 V, (**c**) −100 V, (**d**) −150 V, (**e**) −200 V.

The morphology of the AlCrNbSiTiN coatings is shown in Figure 1. There are a large number of particles on the surface of the coatings deposited without bias voltages, and the surface roughness is too large to be measured by atomic force microscopy. The typical droplet morphology is observed on the surface of the coatings, which is a common defect in AIP. Figure 1 reveals that elevated bias voltages suppress characteristic arc-plating defects and droplet density. This stems from the electrostatic repulsion of negatively charged macroparticles within substrate sheath regions [24,25]. The surface roughness remained stable at around 50 nm (from 50 to 100 V) but plunged to 29.79 nm at 200 V, while coating thickness dropped from 12.43 μm (0 V) to 2.45 μm (200 V) due to resputtering-enhanced densification. The ionic kinetic energy and resputtering effect increase with increasing bias voltages, which leads to the densification of the coatings and the decrease in thickness. The bias voltages have no obvious effect on the proportion of coating elements.

**Figure 2:**
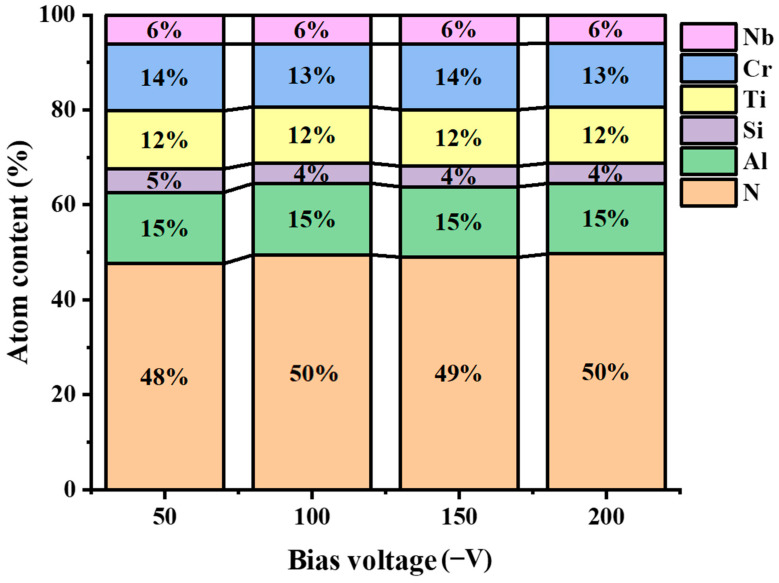
The chemical composition of the AlCrNbSiTiN coatings with different bias voltages.

The chemical composition of the AlCrNbSiTiN coatings with different bias voltages was shown in Figure 2. The proportion of N element does not change significantly with increasing the bias voltage, indicating that the bias voltage has a small influence on its content [26].

### 3.2. Mechanical and Tribological Properties

**Figure 3:**
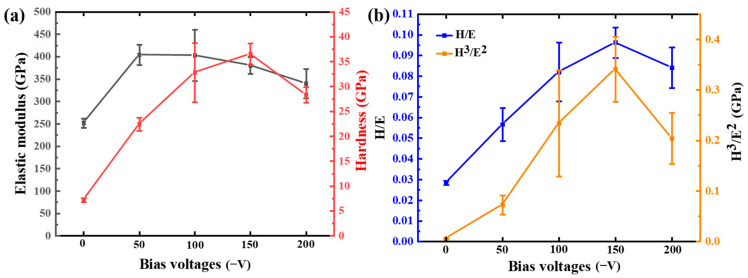
(**a**) Hardness and elastic modulus, and (**b**) H/E and H^3^/E^2^ of the coatings with different bias voltages.

Figure 3a indicates that the hardness of the coatings varies with bias voltages. The elastic modulus tends to decrease gradually as the bias increases. The structure of the coatings becomes denser, and the internal defects reduce with increasing bias voltages, so the hardness increases. As bias voltages rose to 150 V, hardness peaked at 36.5 GPa, which precedes many reported coatings [27,28]. The coatings deposited at −150 V have few internal defects and a dense structure. The coating grains are closely arranged, and the reduction in crystal plane spacing produces a boundary strengthening effect. The re-sputtering effect at −200 V bias voltages increases, leading to more internal defects in the coatings and an increase in residual stress. The coatings deposited with −200 V bias showed a lower performance.

It can be seen from Figure 3b that the variation trends of H/E and H^3^/E^2^ results with bias voltages are basically consistent with the variation in hardness with bias voltages. The H/E value reflects the ability of the coatings to resist elastic strain damage, and the H^3^/E^2^ value indicates the ability of the coatings to resist plastic deformation [22,29]. The greater the H/E and H^3^/E^2^ values of the coatings, the better their toughness. The H/E and H^3^/E^2^ increase from 0.057 to 0.096 and from 0.073 GPa to 0.34 GPa, respectively. The H/E and H^3^/E^2^ at 200 V bias decrease to 0.084 and 0.21 GPa, respectively. The coatings deposited with 150 V bias exhibit the highest hardness and toughness.

**Figure 4:**
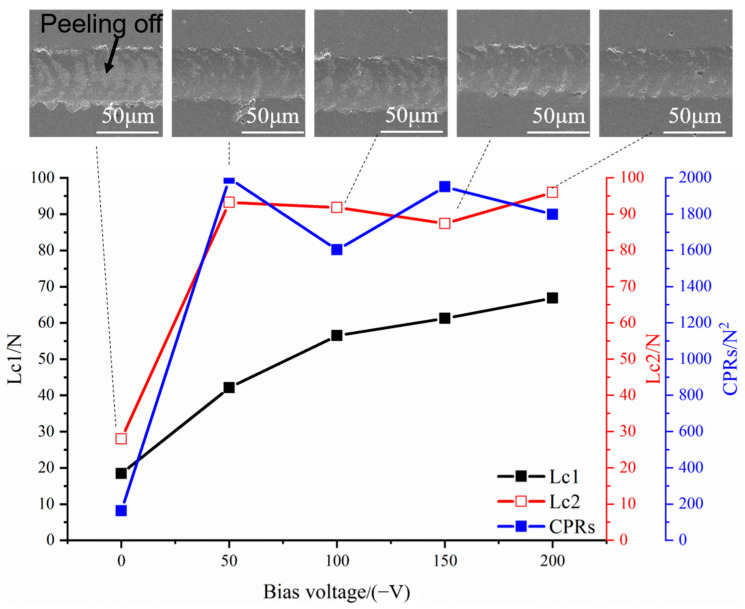
Scratch test results of AlCrNbSiTiN coatings.

The scratch test results of the coatings are shown in Figure 4. The three critical loads *L_c_*_1_, *L_c_*_2,_ and *L_c_*_3_ correspond to three stages in the test process, namely the initial cracking of the coating, the initial peeling, and the complete peeling of the coating. If the bonding strength between the coating and the substrate is high, the third stage may not occur. The adhesion and toughness of the coating can be characterized by the crack propagation resistance value (*CPRs*), which is also known as scratch toughness. Its calculation formula is shown in Equation [30,31]:

*CPR*s = *L_c_*_1_ · (*L_c_*_2_ − *L_c_*_1_)

It can be seen that at 0 V, the coating peels off at 30 N, and its *CPR value* is only 163.3 N^2^. The scratch toughness increased significantly after applying bias. At −50 V, the *CPRs* increased rapidly to 1997 N^2^. With the bias voltage further increasing, the *CPR value* of the coating changed little. The poor scratch toughness at 0 V is mainly attributed to the loose and rough structure of the coatings and the presence of many microscopic defects. When a load is applied, large particles or hole defects running through the entire coating will preferentially form cracks and are prone to extend to the substrate, resulting in premature peeling and failure of the coating. Furthermore, the coating shows residual tensile stress, which is not conducive to inhibiting the propagation of cracks. After applying the bias, the coating structure becomes densified, the microscopic defects are improved, and the residual compressive stress existing in the coating can hinder the propagation of cracks. The particles emitted in AIP have a high displacement rate. These ionized ions have high kinetic energy, which can promote the densification of the growth film, increase the mobility of adsorbed atoms, reduce structural defects in the coating, and improve the quality of the coatings. And it can also increase the surface activity of the substrate and enhance the bonding strength between the coating and the substrate. Both will enhance the scratch toughness of the coatings.

By comparing the results of the scratch test with values of H/E and H^3^/E^2^, it can be seen that the results are not completely consistent. At −50 V, the coating has the lowest H/E and H^3^/E^2^ values, but it has the highest scratch toughness. The reason is that H/E and H^3^/E^2^ have been used as evaluation indicators for the fracture toughness of hard coatings in the past decade, and this evaluation index is well-representative in ceramic coatings [32]. However, studies have shown that in some coating systems, this index is not related to toughness, and even shows an opposite relationship, such as TiB_2_-TiC-Al_2_O_3_ composite coating [33], Fe/VC multi-layer coating [34]. Chen et al. [35] proposed that H/E and H^3^/E^2^ cannot reflect the plasticity and advanced structure in the coating. The coatings do not undergo plastic deformation at fracture, and this indicator can better represent the fracture toughness of the coatings. The coatings undergo plastic deformation at fracture, and this indicator cannot represent the fracture toughness of the coatings. Obvious plastic deformation morphologies were observed in the scratch test of the −50 V specimens, resulting in a decrease in the applicability of the H/E and H^3^/E^2^ evaluation indicators.

**Figure 5:**
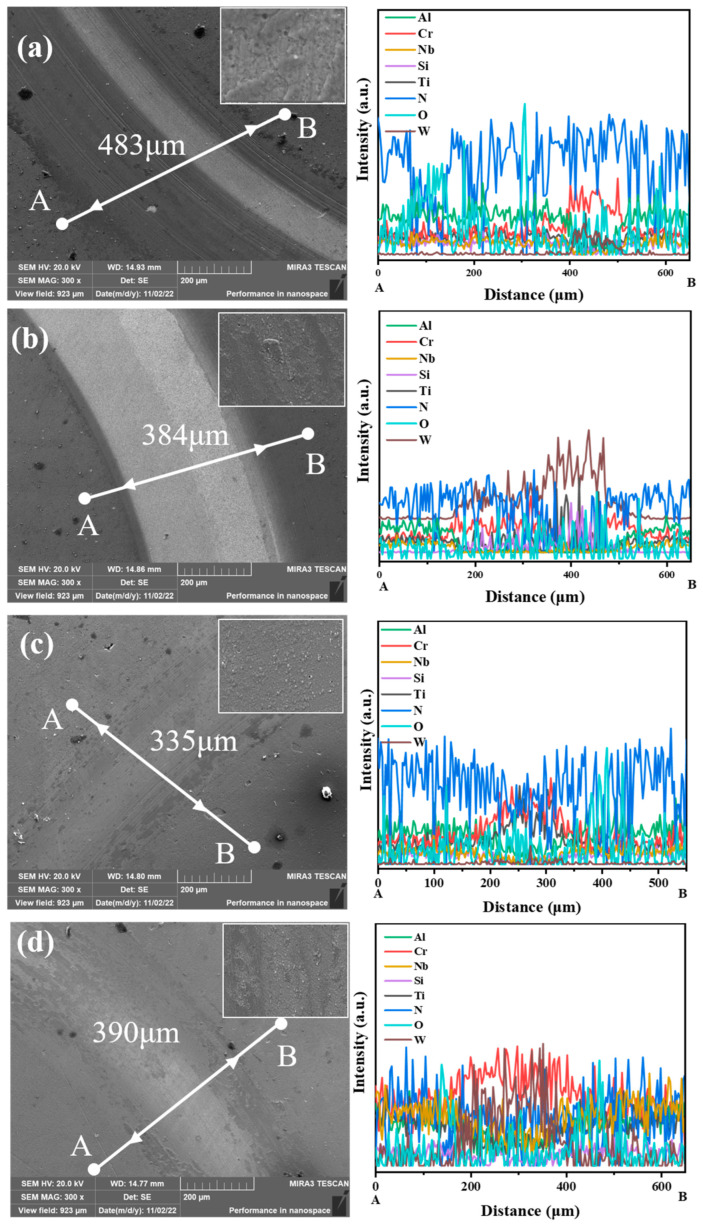
Abrasion marks and EDS results on the AlCrNbSiTiN coating. (**a**) −50 V, (**b**) −100 V, (**c**) −150 V, (**d**) −200 V.

The wear morphology of the coatings is shown in Figure 5. The coatings used for testing were deposited on cemented carbide substrates. According to the EDS results, the Cr element and W element in the coatings at 50 V increased significantly, and the Al, Nb, Si, and Ti elements decreased significantly, which proves the peeling of the coatings. The proportion of W in the coatings with 100 V bias increased significantly, proving that the coatings were completely damaged. The proportions of Cr and Ti elements in the coatings deposited at 150 V increase significantly, while the proportions of Al, Nb, and N elements decrease significantly. There are a large number of furrows parallel to the direction of friction. This is because the oxide wear chips cause local plastic deformation during the friction process, resulting in abrasive wear. The proportions of W and Cr elements in wear marks with 200 V bias increased significantly, while the proportions of Al, Ti, and Nb elements decreased significantly, resulting in local peeling of the coatings. In the edge grinding debris area, the proportion of O element increases significantly. During the friction process, oxidation occurs on the grinding material and the coating. As the friction continues, the oxide film partially peels off, and new oxide films continue to form. This repetitive process leads to the widening of the grinding marks and the continuous peeling off of the coatings. The wear mechanism of the coating is oxidative wear. During the friction process, an oxide layer was formed between the coating and the grinding ball. The oxide layer was damaged under the load to form grinding chips. Abrasive wear occurs between the abrasive chips and the coating. In addition, the grinding ball can cause microcracks in the coating under the load. The formation, expansion, and aggregation of these microcracks can lead to peeling of the coating.

### 3.3. High-Temperature Oxidation Behavior

**Figure 6:**
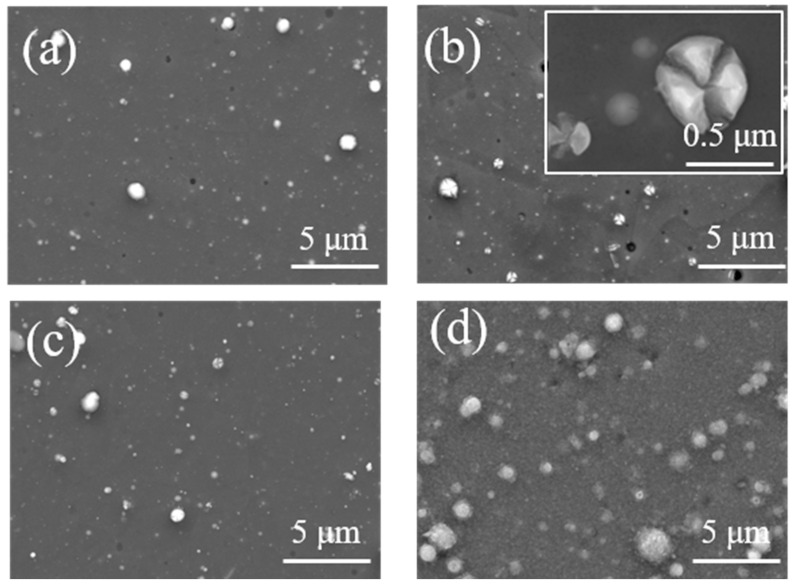
Surface morphology of the AlCrNbSiTiN coatings prepared at −150 V bias voltages after oxidation at different temperatures. (**a**) 600 °C, (**b**) 700 °C, (**c**) 800 °C, (**d**) 900 °C.

The coatings prepared at −150 V bias voltages were annealed in an atmospheric environment. Figure 6 shows the morphology of the coatings after oxidation at different temperatures. After annealing at 600–700 °C, the surface morphology of the coating remains basically unchanged, but some particles on the surface grow larger. Due to excessive internal stress within the particles, cracking occurs. The surface of the coatings begins to oxidize at 800 °C, with fine oxide particles appearing. After annealing at 900 °C, many fine oxide particles appeared on the surface of the coating, and the growth orientation of the oxide particles was not completely consistent.

**Figure 7:**
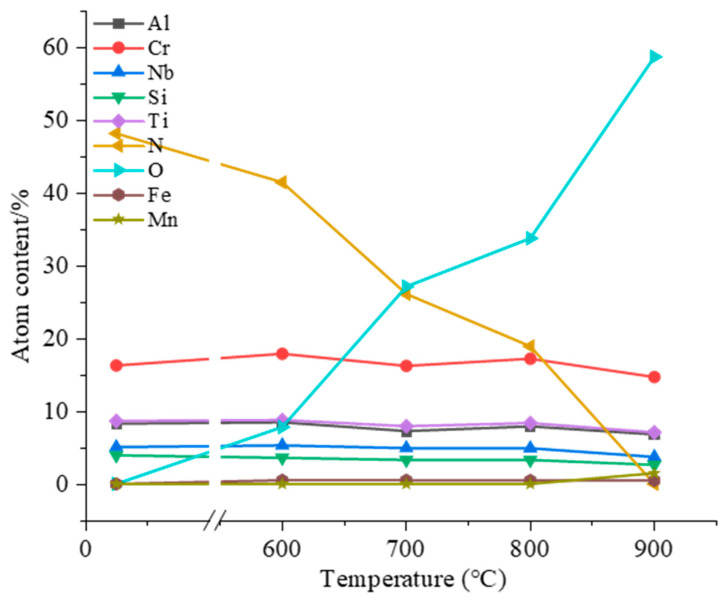
Chemical composition of AlCrNbSiTiN coatings prepared at −150 V bias voltages after high-temperature annealing at different temperatures.

The chemical composition of the AlCrNbSiTiN coatings after high-temperature annealing at different temperatures is shown in Figure 7. As the annealing temperature increases, the oxygen content in the coating keeps rising while the nitrogen content keeps decreasing. After annealing at 900 °C, the nitrogen content in the coating drops to 0, and the coating is completely oxidized. After annealing at 600–800 °C, the content of metal elements in the coating fluctuates slightly, but the change is not obvious. After annealing at 900 °C, the content of metal elements in the coating all decreased, indicating that the metal elements were oxidized to form an oxide layer. As the oxidation proceeded, the oxide layer was destroyed, resulting in a decrease in the content of metal elements. The substrate elements, Fe and Mn, were detected in the coating after annealing at 900 °C, indicating that during the annealing process, mutual diffusion occurred between the coating elements and the substrate elements.

### 3.4. Water Vapor Corrosion Resistance

**Figure 8:**
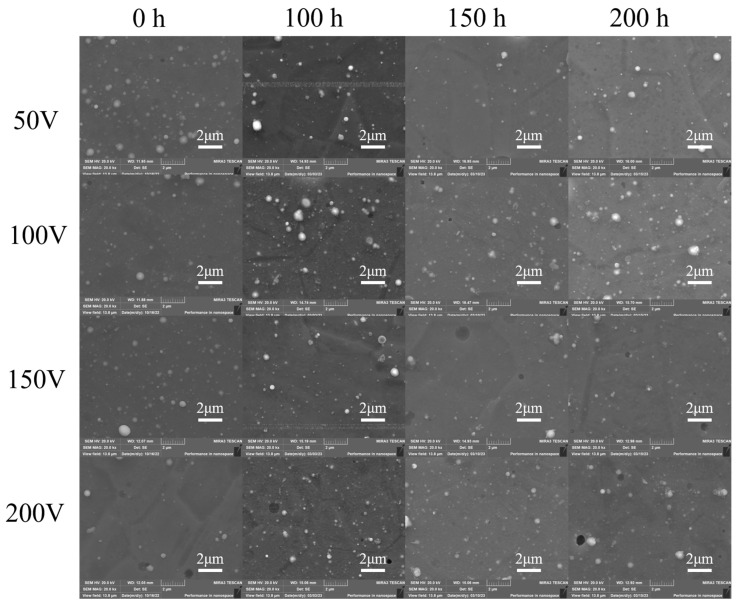
Morphology of AlCrNbSiTiN coatings after water vapor corrosion.

**Figure 9:**
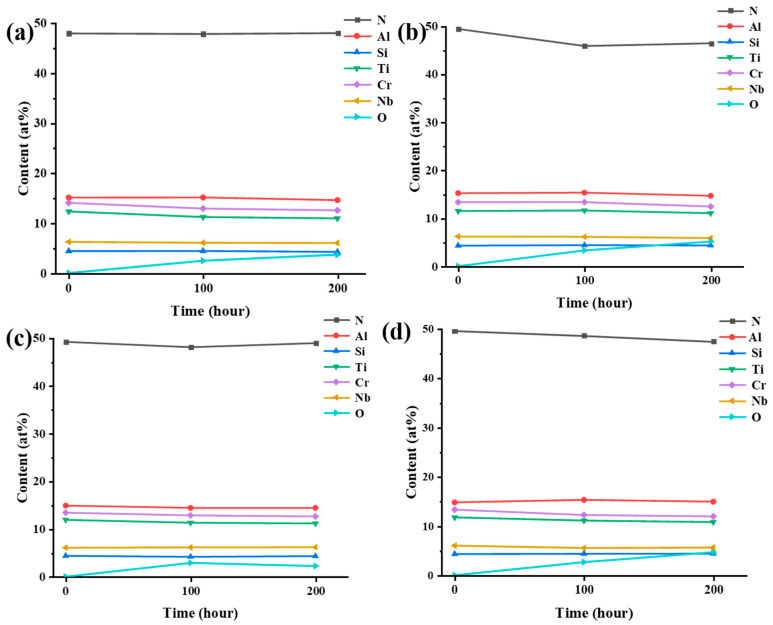
Elemental proportions after water vapor corrosion of the coatings, (**a**) −50 V, (**b**) −100 V, (**c**) −150 V, (**d**) −200 V.

The morphology of the AlCrNbSiTiN coatings after water vapor corrosion is shown in Figure 8. After 200 h of corrosion, the surface morphology of the coatings basically remained unchanged. Figure 9 shows the surface chemical composition of the coatings. The proportion of O element in the coatings under different bias voltages all increased with increasing corrosion time. After 200 h of corrosion, the proportion of O element on the coating surface changes slightly. Among them, the proportion of O element in the coatings at 100 V bias changed the most, being 3.30 at% and 5.15 at%, respectively, after 100 h and 200 h of corrosion. The coatings at 150 V bias had the least change, and the proportion of O element after 200 h of corrosion was 2.23 at%. Due to the high antioxidant performance of nitrides, the corrosion rate of the coating surface is relatively low, which has also been reported in relevant literature [36,37]. Studies have shown that Cr-based coatings significantly reduce the oxidation rate [38]. In addition, the Cr and CrN transition layer in multi-principal element nitride coatings can also enhance the adhesion and corrosion resistance of the coatings [39]. The AlCrNbSiTiN coatings exhibit excellent resistance to water vapor corrosion.

### 3.5. Electrochemical Corrosion Behavior

**Figure 10:**
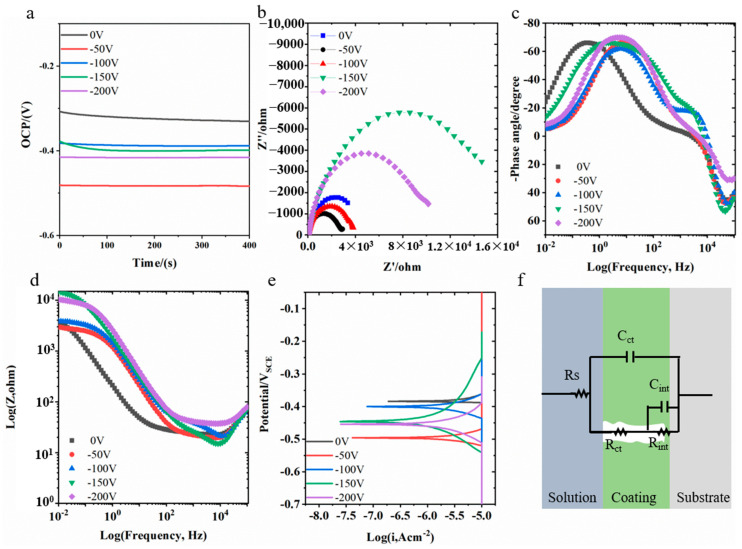
Electrochemical results of AlCrNbSiTiN coatings under different bias voltages: (**a**) Open circuit potential, (**b**) Nyquist plot, (**c**) Bode-phase plot, (**d**) Bode-module plot, (**e**) Polarization curves, (**f**) Equivalent circuit diagram.

Electrochemical results for the coatings are presented in Figure 10. The coatings deposited without bias voltages exhibited the highest open circuit potential (OCP), approximately −0.3 V. After applying −50 V bias, the OCP decreased to −0.48 V. Increasing the bias to −100 V raised the OCP to −0.38 V. Subsequent increases in bias voltages stabilized the OCP around −0.4 V. The Nyquist plots for all coatings displayed imperfect semicircles. At 0 V bias, the coatings had the smallest impedance arc radius. The radius increased after applying a −50 V bias. At −100 V, the capacitive loop radius slightly decreased. When the bias increased to −150 V, the capacitive loop radius significantly increased. Further bias increase leads to a decrease in the capacitive loop radius. The coating prepared at −150 V exhibited the largest capacitive loop radius. The Bode-phase plots indicate that all coatings exhibited two time constants. The high-frequency time constant corresponds to the coating properties, while the mid-to-low frequency time constant originates from the coating/substrate interface. The equivalent circuit model established based on this is shown in Figure 10f. The circuit parameters obtained from fitting using this model are listed in Table 1. The table shows that the coating resistance value (R_ct_) is far greater than the interface resistance (R_int_). Therefore, the primary contribution to corrosion resistance comes from the protective nature of the coating itself. At low bias voltages, the R_ct_ values were relatively small. When the bias voltage increased to −150 V and above, the R_ct_ values increased significantly by an order of magnitude. The sample prepared at −150 V had the highest resistance value, with R_ct_ reaching 1.68 × 10^4^ Ω·cm^2^. The polarization curves of the coatings are shown in Figure 10e. The calculated corrosion potential and corrosion current density are also listed in Table 1. The variation in corrosion current density with bias voltages is consistent with the EIS results. Coatings prepared at bias voltages below −150 V exhibited higher corrosion current densities. Coatings prepared at higher bias voltages exhibited lower corrosion current densities. The coating prepared at −150 V had the lowest corrosion current density, 0.79 μA·cm^−2^.

From the electrochemical corrosion results, it is evident that samples prepared by AIP exhibited poor corrosion resistance without applied bias voltage. This is attributed to the loose structure of the coatings, which accelerates the penetration of corrosive media. After applying bias voltage, AIP-prepared coatings attained superior corrosion resistance at high bias voltages. For AIP coatings, large particle defects are prominent. Therefore, the droplet-like particle defects within the coatings dominate the differences in corrosion resistance under various bias voltages. At high bias voltages, the particle defects are mitigated, leading to superior corrosion resistance.

**Figure 11:**
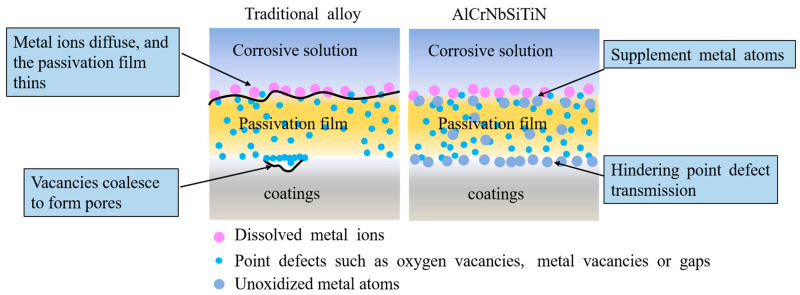
The corrosion mechanism of traditional alloys and AlCrNbSiTiN coatings.

The corrosion mechanism diagrams of traditional alloys and AlCrNbSiTiN coatings are shown in Figure 11. The traditional alloy point defect model holds that there are high concentrations of point defects in the passivation film on the coating surface, such as metal vacancies or gaps, and oxygen vacancies. During anodic polarization, point defects participate in the defect reactions at the metal/passivation film interface and the passivation film/corrosion solution interface through the transport of the passivation film. The aggregation of metal vacancies at the metal/passivation film interface will form pores at the interface and damage the passivation film. Metal ions diffuse from the passivation film to the corrosive solution without receiving sufficient metal replenishment, and the passivation film will become thinner. Under the action of these two mechanisms, the passivation film of the coating dissolves. These atoms that do not participate in oxidation can provide a double-layer protection mechanism during the corrosion process. On the one hand, it can impede the transmission of point defects in the passivation film, enhancing the stability of the passivation film. On the other hand, these atoms are not easily diffused into the corrosive solution through the passivation film, thus supplementing metal atoms to the passivation film and inhibiting its dissolution.

## 4. Conclusions

The structure and properties of the AlCrNbSiTiN coatings were optimized by AIP technology combined with the regulation of bias voltage. By taking advantage of the characteristics of high ionization rate and large ion kinetic energy of AIP technology, the point defects inside the coating have been effectively improved, the internal stress of the coating has been alleviated, and the hardness of the coating has been enhanced. The coatings can achieve a maximum hardness of 36.5 GPa under low stress conditions. Meanwhile, the scratch toughness of the coatings was improved, and their *CPR value* was increased to nearly 2000 N^2^. The wear resistance of the coatings has been significantly enhanced, providing good protection for the substrates. The coatings exhibit excellent resistance to water vapor corrosion. After 200 h of water vapor corrosion, the proportion of O element on the coating’s surface is only 5.15 at%. The coating exhibited corrosion resistance in 3.5 wt.% NaCl solution. Particle defects were the main factor dominating the corrosion resistance of the coatings. At −150 V, the coatings had the optimal corrosion resistance, with the coating resistance and corrosion current density being 1.68 × 10^4^ Ω·cm^2^ and 0.79 μA·cm^−2^, respectively.

## Figures and Tables

**Table 1:** Electrochemical parameters, corrosion potential, and corrosion current density of AlCrNbSiTiN coatings.

	0 V	−50 V	−100 V	−150 V	−200 V
R_s_/Ω·cm^2^	19.47	23.20	21.59	15.14	37.50
CPE_ct_/10^−5^ F·cm^−2^	7.2	7.2	1.1	9.9	3.0
n_ct_	0.92	0.89	0.87	0.91	0.98
R_ct_/10^4^ Ω·cm^2^	0.27	0.44	0.38	1.68	1.00
R_int_/Ω·cm^2^	7.8	2.5	21.8	13.9	8.7
CPE_int_/10^−5^ F·cm^−2^	14.83	115.57	14.21	12.96	7.60
n_int_	0.84	0.82	0.79	0.77	0.85
E_corr_/V	−0.38	−0.49	−0.40	−0.45	−0.45
I_corr_/μA·cm^−2^	3.01	1.62	1.66	0.79	1.25

## Data Availability

The original contributions presented in this study are included in the article. Further inquiries can be directed to the corresponding authors.
